# Bayesian analysis supports the role of alectinib and ensartinib for ALK‐positive non–small cell lung cancer

**DOI:** 10.1111/1759-7714.14877

**Published:** 2023-04-02

**Authors:** Alessandro Rizzo

**Affiliations:** ^1^ IRCCS Istituto Tumori “Giovanni Paolo II” Bari Italy

To the Editor,

In the current systematic review and network meta‐analysis (NMA) published by Peng et al.,^1^ the authors pulled together 12 clinical trials for a total of 3169 patients with advanced‐stage anaplastic lymphoma kinase (ALK) rearrangement‐positive non–small cell lung cancer (NSCLC). According to the results of the NMA, Peng and colleagues^1^ suggested that alectinib may be the most efficient treatment for ALK‐positive NSCLC. At the same time, ensartinib reported a significant progression‐free survival (PFS) advantage over alectinib.^1^

Peng et al.^1^ performed NMA to optimize data extrapolation and to compare different treatments when no direct comparative trial was available and to obtain more precise effect estimated by jointly considering direct and indirect comparisons.^2^ Here, the authors used rigorous, well‐accepted methods to assess evidence across clinical trials, also acknowledging important limitations.

However, we believe some methodological issues would deserve discussion.

First, Bayesian NMA—similarly to pairwise meta‐analysis—may be associated with the inflation of false positive (type 1) and false negative (type 2) errors; because these specific errors have been suggested to play an important role to validating true‐positive as well as true‐negative findings in meta‐analyses, this issue should be carefully considered.^3^

In our view, Peng et al.^1^ are to be commended for this interesting NMA aimed at evaluating a timely topic in ALK‐positive NSCLC. At the same time, NMA has some limitations that should be considered and cannot replace head‐to‐head clinical trials comparison. Based on these premises, the NMA by Bao and colleagues further emphasizes the need for large‐scale, well‐designed clinical trials aimed at investigating ALK inhibitors in this setting.

We invite the authors to share their view on these remarks.

## AUTHOR CONTRIBUTIONS

All authors contributed to the article and approved the submitted version.

## CONFLICT OF INTEREST STATEMENT

The authors declare that the research was conducted in the absence of any commercial or financial relationships that could be construed as a potential conflict of interest.
